# The Library of Medicine

**Published:** 1841-07

**Authors:** 


### Art. VI.

*The Library of Medicine.* Arranged and edited by Alexander Tweedie, m.d. f.r.s., &c. (Practical Medicine.) Five Volumes. —*London*, 1840. 8vo, pp. 440, 353, 385, 361, 346.

The readers of this Journal have already (Br. and For. Med. Rev., vol. X., p. 231,) been made acquainted with the general plan of the "Library of Medicine," and been given some notion of the aim and merits of its two introductory essays—that on General Pathology by Dr. Symonds, and that on Inflammation by Dr. Alison. We now proceed to furnish them with such an analysis of the five volumes devoted to the subject of Practical Medicine as the pretensions of these demand and circumstances permit.

Vol. I. In addition to the two articles just referred to, this volume contains essays on "Primary," Eruptive, and Puerperal Fevers, by Drs. Christison, Shapter, Gregory, Burrowes, and Locock, and concludes with a description of Cutaneous Diseases, by Dr. Schedel.

General Doctrines of Fever: Continued Fever. These papers, contributed by Dr. Christison, will be noticed with other works upon continued fever in our next Number.

Plague. This article is by Dr. Shapter, and is a compilation from which it would be unreasonable to withhold the praise due to judicious selection and arrangement of borrowed materials. The subsequent descriptions of Dr. Shapter are indeed distinguished by sounder intelligence of the subject on the part of their author than the opening paragraph gives promise of. In this, plague is stated to be "an *exanthematous* disease, the eruption consisting of buboes, carbuncles, and pustules, white, livid, or black, and generally attended with malignant and very fatal fever." This definition, which Dr. Shapter bears not the weight of originating, evidently sins in a multitude of manners both logical and critical. We shall only here ask how Dr. Shapter justifies the strange perversion whereby buboes, carbuncles, and pustules are spoken of as the characteristics of an *exanthema?* Medical language is assuredly sufficiently loaded with equivocal terms and ambiguous phraseologies to render any attempt to add to their number, to say the least, superfluous.

The recapitulation of the arguments respecting contagion is well executed. The arguments and alleged facts adduced by the contagionists and their opponents are perhaps more likely to be fairly estimated by those knowing the scourge but by report, than by those who, living within the sphere of its inflictions, have interests, fears, or prejudices to be affected by the decision. Dr. Shapter inclines to believe, upon mature consideration of the arguments of each party, that plague is "essentially an endemic disease;" that through the influence of circumstances hitherto unascertained, but connected with season, it occasionally becomes epidemic; that "during the continuance of the epidemic effect, a principle is given off from the body, which *if very concentrated and pent up in confined and unwholesome situations* may generate the disease, so that, though not originally contagious, it may in that way, by accumulation of animal miasms, become contagious." The disease, then, according to Dr. Shapter, is evidently not contagious in the legitimate acceptation of the term; yet he appears to swerve from this conclusion, by maintaining it to be "not improbable that when the disease is communicated from person to person, it is by the inhaling the pestiferous breath or exhalations which emanate from the body of the patient; but at the same time that this influence of the atmosphere of contagion is very limited in its power and extent. A person who is himself uninfected cannot, according to Dr. Shapter, produce the disease in others by being "as it were the bearer of it." He adds that "the communication of plague by inoculation with the matter from a bubo or with any other morbid product, has by no means been proved; on the contrary, there is every reason to believe that the disease cannot be produced by these means." This is a perfectly correct estimate of experimental results:* persons have been frequently inoculated with the matter of buboes without the disease being produced; and in the instances of the few subjects in whom the occurrence of the malady has followed the operation, the *post hoc propter hoc* argument is inadmissible, inasmuch as they were exposed to the local influences generating the disease, and might have had it independently of the experiment performed.

The chapter upon Intermittent Fever is also the production of Dr. Shapter; and having herein more the tone of an observer, the writer appears to greater advantage than in his other writings. There is nothing in the description of the malady, however, meriting particular notice, except, we regret to say, a very remarkable omission, that of all allusion to the valuable researches of Professor Piorry into the condition of the spleen in intermittent fevers, an omission the less excusable because the results of these researches were some while since transferred to the literature of our own country in the pages of this Journal.† It will be

* Vide Br. and For. Med. Rev., vol. V. p. 560. April, 1838.

† Ibid., vol. VI. p. 141. July, 1838.

sufficient to quote the following statements to prove the importance of M. Piorry's enquiries: "The effect produced by quinine upon tertians and quartans is *proportionate to the reduction of the spleen;* so that the disease is cured simultaneously with restoration of this viscus to its healthy dimensions. On the other hand, though the symptoms be arrested, they will be liable to recur as long as the spleen exceeds its proper size. As the spleen attains its greatest enlargement at an early period of the disease, it proves that *it is not the paroxysms that produce the hypertrophy, but rather the enlarged organ that maintains the disease.*"

Dr. Shapter has been intrusted with the subjects of Remittent and Yellow Fever also. His account of the latter has unfortunately lost the greater part of any interest which might once have appertained to it by the subsequent publication of M. Louis' work on the disease, a work containing, as our readers are aware, the results of the first energetic and well-guided attempt to unravel the obscure anatomy of the malady. Dr. Shapter's essay is, however, well put together, and did it contain nothing else to admire, the subjoined sentence alone would render it deserving of purchase: "In order to prove the agency of contagion, a condition is absolutely necessary, which hitherto has never been properly attended to, viz. that the persons to whom the disease has been supposed to be communicable, should not reside in the same situation or locality as those by whom it is believed to be communicated, as in such case their being subject to the same influences as those already diseased, entirely invalidates any argument that may be offered in support of the operation of contagion." It is not writers on *yellow* fever alone who may be benefited by pondering upon this incontrovertible position.

Dr. Shapter informs us that Sir W. Burnett, Physician General of the Navy, "became so convinced of the absolute necessity of the free abstraction of blood in yellow fever, that he issued orders to the surgeons of the fleet, when stationed in the Mediterranean, to the effect that they should pursue this practice in every case of yellow fever that came under their inspection."* On the other hand, numerous English observers, together with "nearly all the practitioners in the West Indies, and many of the American and Spanish physicians," are cited as affirming, that they who bleed a man suffering under yellow fever "are guilty of nothing short of murder." Now there are certain persons terming themselves "practical men," who sneer at the idea of deciding upon the respective merits of any two given systems of medication by *counting how many die and how many recover under the application of each:* there's no "*genius*" and no "*practical tact*" wanted for an "*addition sum,*" and *they* are all men of genius of course. Will these sages inform us how else than by cautious employment of the numerical method the point at issue between Sir W. Burnett and the other individuals referred to can be set at rest? "We pause for a reply."

* This statement is not accurate as regards Sir William Burnett. He was and is an advocate for bloodletting in certain cases of yellow fever, and often advised and practised it; but he never was an indiscriminate bleeder, and never issued any order enforcing it. —Ed.

Dr. Locock, the author of the article Infantile Gastric Remittent Fever, writes concerning it as follows: "There is apparently a striking confirmation of the modern doctrines of Broussais as to the nature of fever in the acknowledged *cause* of this infantile disease. The most prominent symptoms are referred to the mucous lining of the stomach and intestine; an acute or a protracted form of fever is the *result;* and with an improved condition of the alimentary canal, the febrile paroxysms are mitigated and gradually disappear." Here a certain affection of the gastro-enteric mucous membrane is pronounced to be not only the original cause, but to furnish a measure of the intensity, of the group of symptoms which it has been the custom to term infantile remittent fever; in fact to bear the same relation to his so-called primary fever, as inflammation of the lung to the fever of pneumonia; and all this is most perfectly correct. But in acknowledging the accuracy of Dr. Locock's pathology, we wish he had gone a step further, and denied to the malady the term "fever." The interpolation "gastric" is a concession no doubt; but even this should have been "gastro-enteritic."

The sketch of the disease is practical and to the purpose.

Hectic Fever has fallen to the lot of Dr. Christison. "True hectic," according to Dr. Christison, "occurs only in connexion with serious organic alterations of structure, and seldom [?], unless when suppuration exists." In the same page it is stated that "hectic like other fevers is obscure in the beginning, and can scarcely be distinguished from the febrile state . . . . which attends some chronic internal inflammations, and chronic visceral derangements of structure." Why, we had thought from sentence the first that hectic *was* the fever attending chronic visceral derangements of structure! The attempt made in this article to describe hectic fever as a distinct entity, to make out the conditions of the various functions during its existence to be dependencies upon it, instead of, as common observation and experience prove them to be, coincident effects of the original organic lesion, or results of secondary changes of structure developed during the progress of this, is, we humbly submit, altogether a misconception. To what purpose have such men as Louis, Andral, and Carswell laboured, if their close inferences respecting the organic causes of functional aberration are thus to be set at nought? To what purpose has the science of medicine made advance, if our students are to be still taught these antiquated notions, characteristic of an era when pathological anatomy was yet unknown? Among the "symptoms" of "hectic fever" here enumerated, is "crookedness of the nails." It might be too critical to remark that, in strict language, such a phenomenon, if it existed, ought to be denominated not a symptom but a sign; but it is important to state that the notion itself has been shown to be fallacious by the investigations of M. Vernois, the first that were ever undertaken upon correct principles in regard to this point.

Smallpox. This article is by Dr. Gregory, and gives a clear, well-arranged, and neatly-condensed account of the existing state of knowledge respecting this important disease, and the allied subjects of inoculation and vaccination.

The two subjoined tables are extremely valuable.

*Table exhibiting the comparative Mortality in the several Varieties of Normal and Abnormal Smallpox at the Smallpox Hospital during the Epidemic of* 1838.

| | UNPROTECTED. | | VACCINATED. | |
|---|---|---|---|---|
| | Admitted. | Died. | Admitted. | Died. |
| NORMAL SMALLPOX. | | | | |
| *Confluent* ............ ... .... | 295 | 149 | 56 | 21 |
| *Semi-confluent* ............. | 78 | 8 | 42 | 4 |
| *Distinct* ..................... | 19 | 0 | 20 | 0 |
| Total Normal...... | 392 | 157* | 118 | 25 |
| ABNORMAL SMALLPOX. | | | | |
| *Confluent modified* ........... | 2 | 0 | 38 | 4 |
| *Semi-confluent modified* ... | 1 | 0 | 28 | 1 |
| *Varicelloid* .................. | 1 | 0 | 114 | 1 |
| Total Abnormal ... | 4 | 0 | 180 | 6 |
| Grand Total ...... | 396 | 157 | 298 | 31† |

* Of these there died of fever and superadded erysipelas 14.
† ,, ,, disease . 10.

*Table exhibiting the Mortality of Smallpox at different Ages and under different circumstances, as displayed at the Smallpox Hospital in the Epidemic of* 1838.

| | UNVACCINATED. | | VACCINATED. | |
|---|---|---|---|---|
| Ages. | Admitted. | Died. | Admitted. | Died. |
| Under 5 | 42 | 20 | 0 | 0 |
| 5 ... 9 | 37 | 11 | 5 | 0 |
| 10 ... 14 | 30 | 8 | 25 | 0 |
| 15 ... 19 | 104 | 32 | 90 | 6 |
| 20 ... 24 | 115 | 50 | 106 | 16 |
| 25 ... 30 | 45 | 23 | 55 | 8 |
| 31 ... 35 | 12 | 7 | 13 | 1 |
| above 35 | 11 | 6 | 4 | 0 |
| Total... | 396 | 157 | 298 | 31 |

It appears from the first of these tables that during the epidemic of 1838, the total mortality among those admitted was 27·1 per cent.; among the unprotected 39·6 per cent.; among the vaccinated 10·4 per cent.

The only omission of any consequence which we have detected in the essay, occurs under the head of mortality; no notice is taken of the original and invaluable researches of Mr. Farr (Brit. Med. Almanack, 1838,) into the law of death and recovery in different stages of the disease. We have, however, to acknowledge an obligation to Dr. Gregory for the table transcribed beneath, which, as he observes, "points out how little importance can be attached to the doctrine of critical days in smallpox."

Of 168 cases of smallpox there died on the

**First Week.**

| Days. | Cases. |
|---|---|
| 3d | 1 |
| 4th | 5 |
| 5th | 10 |
| 6th | 5 |
| 7th | 11 |
| | 32 |

**Second Week.**

| Days. | Cases. |
|---|---|
| 8th | 27 |
| 9th | 15 |
| 10th | 14 |
| 11th | 16 |
| 12th | 11 |
| 13th | 11 |
| 14th | 5 |
| | 99 |

**Third Week.**

| Days. | Cases. |
|---|---|
| 15th | 7 |
| 16th | 5 |
| 17th | 3 |
| 18th | 3 |
| 19th | 1 |
| 20th | 2 |
| | 21 |

**Fourth Week and later.**

| Days. | Cases. |
|---|---|
| 22d | 3 |
| 23d | 1 |
| 24th | 3 |
| 25th | 1 |
| 27th | 1 |
| 28th | 1 |
| 29th | 1 |
| 31st | 1 |
| 32d | 1 |
| 35th | 1 |
| 38th | 2 |
| | 16 |

Dr. Gregory exhibits himself, to a certain extent, the apologist of inoculation. The culminating objection to inoculation is, as all are aware, that it perpetuates and multiplies the sources of contagion. Certain considerations adduced by Dr. Gregory, "must," he believes, "convince any unprejudiced mind that the argument against inoculation, drawn from its supposed tendency to augment and multiply the force of contagion, is not so forcible as the opponents of contagion invariably allege." One of these considerations is that "in 1838, when inoculation was unknown in London, the admissions into the Smallpox Hospital exceeded those of 1781, when inoculation was universally practised; both being years of epidemic prevalence." As it stands, this proposition is valueless; Dr. Gregory should at least have shown that the increase was out of proportion with that of the population.

"Whether inoculation," observes Dr. Gregory in conclusion, "be ever destined again to occupy the thoughts of men, and to cooperate with vaccination in the general design of mitigating the severity of smallpox, is a question which at the present time it would be certainly premature and perhaps unnecessary to consider." (p. 321.)

The influence said to be exercised by atmospheric constitution over the powers of resistance to variola is regarded by Dr. Gregory as indubitably real: "the number of persons attacked during epidemic seasons, who had successfully resisted smallpox contagion *communibus annis*, offers an argument in favour of the position which to our minds is irresistible."

In respect of recurrence to the cow for primary lymph, Dr. Gregory is "bound to acknowledge that the Smallpox Hospital of London changed their old stock of lymph for more recent matter in 1837, and that a marked improvement was perceived in the resulting vesicles. The local inflammation was more severe; the constitutional symptoms were more violent; the virus was more energetic; the most minute incision took effect, and the lymph given out on the ninth and tenth day was still in an active state."

The phenomena of vaccination are well described, and very useful hints given respecting the mode of practising and conducting the operation.

Revaccination may, according to Dr. Gregory, "be recommended for its safety, even if it be much less serviceable than the Germans contend for. We have sufficient facts before us to state with confidence that it need never be recommended prior to the tenth year of life, and that the age best fitted for it is from the period of puberty to that of confirmed manhood."

Measles and Scarlatina are described by Dr. G. Burrowes. There is little in these descriptions calling for particular notice. "The season of the year" is said to have "a more important influence on the issue of measles than of scarlatina or variola;" a position plausible enough, but requiring numerical demonstration, which is not furnished by Dr. Burrowes.

Under the head "*anatomical characters,*" Dr. Burrowes, as do all the contributors to the volume, enumerates every change of structure discovered in the bodies of persons dying with the particular disease under consideration. This proceeding evinces very curious misapprehension of the proper meaning of the term *anatomical character*, which in reality applies to the especial organic characteristic, and to this alone, of any given malady. Thus, gray or red hepatization is an anatomical character of pneumonia, because the discovery of such lesion on the dead body proves that that disease has existed during life; but softening of the mucous membrane of the stomach is not an anatomical character of, though it is frequently found in subjects dying with pneumonia, simply because it is a change observed in the victims of all varieties of disease. According to the system pursued in this volume, (we give a ludicrous but really a correct illustration, the more forcibly to convey our meaning,) if a man having stricture of the urethra is thrown from his horse and breaks his neck, thickening of the walls of the urethra and diminution of its caliber should be enumerated among the "anatomical characters" of fracture of the spine. It is so uncommon for a number of medical writers to display such perfect unanimity, that we cannot help fancying the individual authors are not justly chargeable with the error pointed out, but that its prevalence is the result of a general order from head-quarters.

Puerperal Fevers. By Dr. Locock. This is a valuable article, and will be examined elsewhere in conjunction with another upon the same subject appearing in the sixth volume of the collection.

Diseases of the Skin. Dr. Schedel has described these affections with perspicuity and conciseness.

Since the satisfactory discovery of the person of the *acarus scabiei*, and of its "local habitation" at the distal point of the furrow communicating with the vesicle, the great therapeutical problem has been how to annihilate the animal with the greatest certitude and dispatch. M. Albin-Gras has made several experiments in search of a sufficiently murderous drug, and finds that the acarus "lives sixteen hours in vapour of burnt sulphur; three hours in water; two hours in olive oil; one hour in the acetate of lead; twenty minutes in vinegar and spirits of wine; twelve minutes in a solution of sulphuret of potash; and only from four to six minutes in a solution of hydriodate of potash." The latter is evidently the *acaricide par excellence*, and probably the best local application; half a drachm of the salt may be mixed with an ounce of lard. We say probably, because it does not appear a necessary consequence that the substance which proves most rapidly destructive of the insect's life when acting on it directly, shall be the most active in attacking it in its habitation. And as M. Schedel observes, "it remains to be explained [although the serosity of the vesicle will not produce the disease and the acarus immediately does,] why the itch is so easily caught by only touching the hand of a person infected with it, for it is difficult to extract the insect furrowed under the cuticle."

Vol. II. The second volume of this work is prefaced by some general observations on the Anatomy, Physiology, and Pathology of the Nervous System, from the pen of Dr. Bennett. These are as comprehensive as the space assigned to them would admit; but this space is so small, that no power of condensation would have enabled the writer to do justice to his subject. There is a physiological inaccuracy which we notice the more particularly because it occurs in the statement of a doctrine of considerable importance, which has afforded more than sufficient matter for dispute and altercation, and with reference to which, therefore, it is essential that everything should be laid down as clearly as possible. The view given by Dr. Bennett of the "excito-motory function" of Dr. Marshall Hall, would convey the notion that in the various instances of this kind of action, the *impression*, though distinct from sensation, is made on the extremity of a sensatory nerve; and that the *motory impulse*, though unconnected with volition, is propagated along the course of an ordinary motor nerve. Now this is not Dr. Hall's doctrine. His doctrine is that, independently altogether of the sensatory and motory tracts of the spinal cord, there is, wrapped up in the same sheath and centrically situated with regard to them, a distinct organ devoted to the excito-motory functions; and that, altogether independent of the sensatory nerves connected with the posterior columns and the motor nerves connected with the anterior columns, there are certain *incident nerves* enveloped in the same neurilemma with the sensatory, by which impressions are conveyed to the excito-motory centre, and certain *reflex nerves* enveloped in the same neurilemma with the motory, by which impulses resulting from such impressions are conveyed to the muscles and there cause contraction.

The general pathological views developed by Dr. Bennett are based on the hydraulic principle maintained by Kellie, that the absolute quantity of blood contained in the cerebral vessels is always the same, however much it may vary in the other parts of the vascular system; but that the relative proportion of blood in the arteries and veins of the brain, as well as the degree of pressure exerted by it, are subject to frequent and rapid variations. Of the general truth of this principle, and of its applicability to pathology, we have no doubt; but we think our author is carrying its application a great deal too far, when he assumes that all derangements of the cerebral functions, the cause of which cannot be explained by structural lesions detected after death, are to be referred to cerebral congestion. (p. 9.)

We admit that we are in no better condition directly to disprove this opinion than Dr. Bennett is to establish it. Facts are wanting; but where this is the case, we are justified in resorting to the most probable analogies. Now, there is every reason to believe that various disturbances of function may occur in a portion of a nerve quite independently of disease of the brain or spinal cord, as neuralgia and paralysis; and it will not be denied that, in many such cases, no derangement of vascular action or lesion of structure can be discovered by the nicest examination; but here the hydraulic principle does not apply, the free tract of the nerve being quite differently related to atmospheric pressure from the inclosed mass of the encephalon;* we are therefore led to admit the possibility of deranged nervous function independently of vascular congestion; and if in one portion of nervous matter, why not in another? If, for example, in the radial or anterior tibial nerve, why not in the brain or spinal cord?

The truth is, we know literally nothing of those molecular changes from which the healthy actions of the nervous system most probably result; and while we continue thus ignorant, we must not hope to arrive at any satisfactory explanation of those morbid actions of the same system which leave no visible traces behind them.

The article on Inflammation of the Brain, by Dr. Hope, has great merit, and evidently embodies the results of much reading and of careful and extensive personal observation. He dwells at considerable length on the precursory symptoms of inflammation of the brain, a subject of vast importance, but of which the investigation is beset with theoretical and practical difficulties.

Dr. Hope distributes his observations under the following heads: 1. Cerebral determination and congestion. 2. Cerebral congestion from debility and inanition. 3. Cerebral irritation, determination, and congestion in infants and children.

A preternatural flow of blood to the brain in consequence of increased arterial action is denominated "cerebral determination." When this exceeds a certain point, the blood, not being carried off by the veins as rapidly as it is introduced by the arteries, accumulates in the latter vessels and produces the state called "active cerebral congestion." When an accumulation of blood takes place in the brain, not from increased arterial propulsion, but from deficient contractility in the capillaries, or from a mechanical impediment to the return of venous blood from the head, the morbid state induced is called "passive congestion," and its seat is principally in the veins and sinuses.

Dr. Hope adopts the hydraulic principle just now adverted to as the groundwork of his reasoning on the derangements of the cerebral circulation, and states that he entertains no doubt of its general accuracy, though he thinks it probable that future experiment will prove the brain to be capable of admitting a small addition to its ordinary quantity of blood. In this we concur, and believe that the principle in question may be safely applied in all reasoning on morbid changes in the circulation within the head: we object to it only when brought in hypothetically to

* We use the word *encephalon* in accordance with the ordinary practice of medical writers, although the proper word is *encephalos;* the former is in reality an accusative improperly used for the nominative. Vide Blaucardi Lexicon ed. Kühn, or Hooper's Dictionary by Grant.

explain cases in which we have no distinct evidence of any change in the circulation, and where the phenomena, for anything we know, may have no immediate dependence on vascular action.

Dr. Hope thinks it may be stated in general terms:

"1. That the symptoms of active determination are those of excitement or exaltation of the cerebral functions; since there is increased arterial action, yet not congestion enough to obstruct the circulation and occasion the opposite train of symptoms. 2. That the symptoms of active or arterial congestion are those of depression of the cerebral functions; because, here the disease has proceeded to the extent of occasioning an obstruction to the circulation through the brain. 3. That the symptoms of passive or venous congestion are likewise those of depression of the cerebral functions; because, here also, there exists an obstruction to the circulation. Now the three states in question are often the precursors both of inflammation of the brain in any of its forms, and of apoplexy and palsy; nor can we always determine in individual instances, why they conduce to the one rather than to the other; but we think it true, as a general rule, that increased determination, implying augmented arterial action, is equally productive of inflammation and of apoplexy and palsy; while congestion, especially the passive form, is more allied to apoplexy and palsy than to inflammation. Sometimes the nature of the exciting cause determines whether the premonitory symptoms shall issue in inflammation or in apoplexy: thus, stooping may occasion apoplexy, while mental excitement may induce inflammation. So formidable are these consequences, whether of one kind or the other, that the states conducing to them must be looked upon as serious maladies; and it is important to the student to know that, in practice, he will encounter an incomparably greater number of 'determinations' and 'congestions,'—in other words, of precursory symptoms,—than of actual inflammations, apoplexies, and palsies." (pp. 11-2.)

With respect to the author's ensuing remarks on the symptoms of these states, as well as on the semeiology of cerebral diseases in general, we think the practical reader will agree with us in regarding them as too explicit and minute to be valid in the present state of our knowledge. At the same time, as they appear to be the fruit of much personal addiction to the subject, they are entitled to a careful perusal.

The description of actual inflammation of the brain and its membranes is very complete and masterly. In treating of meningitis the author enquires whether the arachnoid or the pia mater be the more frequent seat of inflammation; and concludes, in opposition to the opinions of Lallemand, Parent-Duchatelet, and Martinet, that the pia mater is most frequently affected. He states that he adopts this opinion not only from extensive observations of his own, but from an analysis of the cases recorded by several distinguished pathologists; and he agrees with the majority of English writers that we have no means of distinguishing the symptoms of arachnitis from those of inflammation of the pia mater, and that it is therefore expedient to treat of inflammation of these membranes under the common name of *meningitis*. The question whether the symptoms of meningitis can be distinguished from those of cerebritis is discussed by Dr. Hope with great judgment. He admits that meningitis cannot be supposed to exist without exciting inflammation or irritation of the surface of the brain, because the membranes and the contiguous substance of the brain are immediately supplied by the same blood-vessels, which, ramifying and subdividing with extreme minuteness in the membranes, penetrate the cerebral substance in every direction. This arrangement, as observed by M. Georget, constitutes an exception to the ordinary manner in which blood-vessels enter the substance of organs; for these in general being more or less spongy and areolar, the vessels penetrate them by trunks and branches, and the whole of their vascular system exists in their interior: but the brain is not spongy and areolar; it contains no cellular substance; and presents therefore a necessary peculiarity in the distribution of its blood-vessels. Dr. Hope observes that the connexion between inflammation of the membranes and inflammation or sympathetic irritation of the adjacent cerebral surface, which is implied in such a distribution of the blood-vessels is strongly corroborated by morbid anatomy, and no less so by the symptoms of disease, since the lesions of the intellectual, sensitive, and voluntary powers which accompany meningitis argue a disturbance of the functions of the cerebrum itself. Is it then possible to distinguish meningitis from cerebritis during life? Many eminent writers, among whom are Abercrombrie and Georget, believe that it is not possible. Dr. Hope's observations and dissections have led him to a view of the subject which appears to us so just that we shall give it in his own words:

"When we place, on the one hand, meningitis with the least possible degree of inflammation of the surface of the brain, and on the other, cerebritis not implicating the membranes, the difference between the symptoms is so marked, that the diseases can scarcely fail to be distinguished from each other by a discerning practitioner. But when the two affections coexist, the one will so far modify the other, as in a great measure to neutralize the characteristic symptoms of each. Yet the compound or intermediate character of the symptoms in such cases will sometimes indicate even the double affection, and a predominance of the one or the other may occasionally be inferred from the preponderance of its particular symptoms. We are far, however, from supposing that these latter distinctions can be formed with certainty. The utmost length to which it is possible to go, is to establish more or less strong probabilities. Entertaining these views, we shall treat meningitis and cerebritis as distinct affections, and endeavour to point out under each the manner in which it is modified by the coexistence of the other. In this way, the compound affection which is of far more frequent occurrence than either in the independent form, will, we think, be rendered more intelligible than if it were treated separately as a distinct variety. In this view we are countenanced by an analogy derived from the lungs. Pleurisy and peripneumony are always treated of separately; whereas pleuro-peripneumony is not." (p. 29.)

The precepts for the treatment of the various forms of encephalic inflammation are such as are sanctioned by the best experience. The writer seems to place more reliance on mercury in acute inflammation of the brain, than our own observation has led us to do. We quite agree with him, however, on the expediency of using it early: he observes with truth that the fear of its acting injuriously as a stimulant, which is unfounded, frequently prevents its employment till the occurrence of effusion or disorganization has rendered the probability of benefit from its use extremely small.*

* We believe that the fear of the stimulating effects of mercury in acute inflammatory diseases is generally without foundation. When we consider that inflammation is accompanied either by *diminished* or *vitiated* secretion, while the effect of the mercurial erethism is to *increase the natural secretion* throughout the system, we are led to infer that the mercurial erethism is much more likely to supersede than to augment inflammatory excitement.

His estimate of the success of treatment in the chronic forms of cerebral inflammation appears to be somewhat higher than general experience will warrant. He says,

"Great perseverance is often requisite to effect a complete cure of these chronic affections. If the practitioner proceed, unwearied and undaunted for many consecutive months, his efforts will not unfrequently be crowned with success; but if his patience fail after a few weeks, and he be tempted from the apparently low state of the patient to put him on full diet, failure and disappointment will commonly be the reward of his want of firmness." (p. 61.)

We believe that the protracted duration which gives scope for perseverance affords of itself almost the only ground of hope; for in the few cases in which we have had the good fortune to witness a favorable issue, this has appeared to us to have been referrible to the gradual wearing out of the disease much more than to the influence of remedies.

The subject of Hydrocephalus is treated in an able and practical manner by Dr. Bennett. We find no ground of dispute with him and no deficiency which any observation of ours could supply. Pass we therefore to the article Apoplexy, by the same gentleman.

In theorizing on the proximate cause of apoplexy Dr. Bennett's reasoning is in conformity with those views of the peculiarities of the cerebral circulation already several times alluded to, and we need not here reiterate the grounds on which we believe that he has carried the application of those views too far. In speaking of the treatment of apoplexy, Dr. Bennett recommends stimulants in the asthenic form more decidedly than is usual with writers of the present day.

We confess that, in our inmost mind, we have often questioned whether the diversity of treatment founded on the old distinction of apoplexy into sanguineous and serous,—false as that distinction was,—may not have led to better success in practice than the almost uniformly antiphlogistic course pursued of late years. That apoplexy, in its purest and most intense form, may exist without effusion, or any visible change in the brain or its vessels, is we conceive sufficiently proved by the researches of Abercrombie and others; and we think that the recognition of this fact should lead us carefully to reconsider the principles on which we have been accustomed to treat this disease. In the asthenic form, with a feeble pulse and a collapsed state of the system, we can see no reason why stimulants might not be generally beneficial; whether with Dr. Bennett we regard such state as one of cerebral congestion from debility of the capillaries, or whether we ascribe it, as we think may with more probability be done, to a molecular change in the nervous mass, the nature of which is inscrutable in the present state of our knowledge.

The article Insanity, by Dr. Prichard, contains a useful digest of the opinions elsewhere stated more at large by that justly distinguished author. The subject has so recently occupied our attention, that it would be superfluous to recur to it at present.

The article on Delirium Tremens, by Dr. Bennett, condenses the greater part of what is known on the subject: we think, however, the writer has made a serious omission in passing over entirely without notice, the opinion of Broussais that delirium tremens is symptomatic of gastro-enteritis. This opinion, however, untenable as an exclusive one, has been so modified by Dr. Stokes of Dublin, as to assume an aspect of sufficient probability to entitle it to serious consideration. This pathologist believes that where the disease supervenes on a debauch, the case is in effect one of gastritis, accompanied and entirely represented by a cerebral affection ; but that where it arises from the sudden abstraction of the accustomed stimulus, it is to be regarded as a purely nervous disorder. This view of Dr. Stokes's appears to have been well supported by dissections and the results of treatment.* However this may be, the distinction between the two forms of delirium tremens,—that succeeding to a debauch, and that arising from privations of an accustomed stimulus,—is beginning to be generally admitted by intelligent practitioners as an important guide to the treatment, and as such it is properly insisted on by Dr. Bennett.

The succeeding chapter, on CEPHALALGIA, is by the same writer. He distinguishes headach into the following forms :

"The *congestive*, from congestion occasioned by increased or diminished vital action of the heart and blood-vessels. 2. The *inflammatory*, from inflammation of the membranes or substance of the brain. 3. The *sympathetic*, from disorder of the digestive, biliary, uterine, urinary, and other organs. 4. The *organic*, from structural change of the bones of the cranium, or the membranes, or substance of the brain. 5. The *neuralgic*, from affection of the nerves distributed to the integuments. 6. The *metastatic*, from the metastasis of disorders. 7. The *intermittent*, occurring at stated periods." (p. 155.)

We have no objection to this classification of headachs, nor do we doubt the existence of that termed *congestive* ; but we cannot at all agree with our author when he says "it is very probable that every species of headach, except the organic and neuralgic, depends upon a greater or less degree of congestion of the vessels of the brain."

The chapter on EPILEPSY, also by Dr. Bennet, is a good and practical one. It is much to be deplored, however, that the everlasting "cerebral congestion" comes in once more as an indispensable condition to the epileptic paroxysm. Dr. Bennett admits the hereditary character of the disease, in which we fully agree with him ; and we think that the only rational mode of accounting for it is by the supposition of a transmissible peculiarity of organization.†

The article on CATALEPSY AND ALLIED AFFECTIONS, also by Dr. Bennett,

* See Brit. and For. Med. Rev., No. XVIII, p. 477.

† On this and parallel subjects, we think the observations of Dr. Holland, published in his "Medical Notes and Reflections," are highly worthy of attention. The fact that inveterate epilepsy is sometimes met with in persons otherwise perfectly healthy, appears strongly to favour the notion of a peculiarity of structure. We have at present under our care a youth about eighteen years of age, who presents a remarkable example of this. He has been affected with severe and frequent attacks of epilepsy for the last eight years. In all other respects he is in the most perfect health. He is of a robust and powerful frame without plethora, and all his functions are well and vigorously performed. We can assign no cause for his affliction but that it is in his family. His sister, who died at the age of nine years, was epileptic and idiotic, and a brother, who is now living, is epileptic and maniacal. Neither of his parents is subject to any cerebral disease, but his father's mother was epileptic.

is a short one, but long enough, nevertheless, to afford space for certain hints concerning "cerebral congestion," whereat we do not wish to conceal our affliction. Otherwise it contains nothing requiring comment.

The article on Spinal Irritations is from the same hand. Although we think that more than enough has been said of late about spinal irritation, considered as a distinct and peculiar disease, we quite concur in the following remarks of Dr. Bennett:

"In all cases of neuralgia, rheumatism, and hysteria, the spine should be examined; while perhaps there is scarcely a functional disorder to which the young female is liable, which may not occasionally be found connected with spinal irritation. We have often had an opportunity of observing the manner in which numerous disorders have been traced to this source, and feel assured that if practitioners in general would pay greater attention to this complication, many of the extraordinary and anomalous cases which are at present the cause of great embarassment in practice, might terminate in the speedy relief of the patient, and increased credit of the physician." (p. 188.)

In truth we continually meet with cases which are pronounced "anomalous," but which so far from being really so, are simple examples of the well-known pathological law that the effects of irritation at the origin of a nerve may be felt principally at its distal extremity.

On the nature of the morbid state of the spinal cord in the affection under consideration, Dr. Bennett remarks:

"As spinal irritation uncomplicated with other disease rarely terminates fatally, the exact anatomical characters of this affection are unknown. There can be little doubt, however, that in the majority of cases the symptoms are referrible to a state of congestion of the spinal cord or its investing membranes. Ludwig and J. P. Frank have alluded to the effects of spinal congestion and the anatomical circumstances which favour its occurrence. The latter, in particular, has pointed out the absence of valves in the spinal vessels, together with their peculiar distribution on the surface of the cord. Their anatomical structure and arrangement render them peculiarly liable to congestion, as the venous blood must ascend in opposition to gravity. They are also equally pressed on by the cerebro-spinal fluid in a state of health, and any cause which tends to increase or diminish its normal quantity, may readily be conceived to produce venous congestion. Hence derangements in the menstrual function, and the various causes which have been mentioned frequently occasion dorsal and lumbar pains, with other symptoms of spinal irritation. The vessels being principally superficial, unless very much dilated, occasion only partial pressure, and consequently increased action followed by the principal phenomena of this affection, viz. neuralgic pains. That motion is not so commonly increased or diminished, may be attributed to the relation of the cord with the osseous walls which surround it, as pointed out by Ollivier, for the anterior columns are almost immediately applied to the bone, while the posterior are five or six lines distant from it. Independent of any positive evidence, therefore, it may be said that the theory of congestion is fully capable of explaining the phenomena, and is the morbid condition which, of all others, we should expect to follow the known causes of the disorder. On the other hand, we cannot with some authors suppose it to be chronic inflammation, for the changeable nature of the affection and its sudden appearance and disappearance are opposed to such an opinion. With regard to the spinal tenderness, it has been well pointed out by Mr. Locock, (Edin. Med. and Surg. Journal, No. cxxxvi.) that 'an inspection of the vertebral column in an anatomical subject will show at once how impossible it is to press the cord or the nerves going from it in the slightest degree.' We cannot think with him, however, that 'tenderness of the spinal marrow is a sign of little value,' as numerous cases prove that there is a connexion between the situation of the local tenderness and other symptoms, while local treatment has dissipated the disorder, when remedies directed to the removal of its more remote effects have failed." (p. 187.)

With respect to the pain excited by pressure on the spine, it should be remembered that, although the cord is evidently exempted from the immediate effects of the pressure, it is contained in an elastic osseous and cartilaginous tube, the parts of which are moveable on each other, and surrounded by a fluid, through the medium of which vibrations may be easily communicated. We need be at no loss, therefore, to account for the effect of external pressure when the cord is morbidly irritable. We may remark also that a slight tap will often excite more uneasiness than a considerable pressure more slowly applied, which, we apprehend, is owing to the vibration thus occasioned. We had some time ago a female patient in whom we could at will produce a sensation like that of an electric shock, followed by immediate syncope, by striking with the fingers on any part of the spine.

Dr. Bennett mentions, as the most frequent causes of spinal irritation, "uterine disorder; exposure to cold and moisture; dyspepsia, worms, and other sources of irritation in the alimentary canal; affections of the liver, mental emotions; erysipelatous, rheumatic, and eruptive fevers; local injuries, &c." There is one cause which we would specify, because we have observed it to be frequent, namely protracted leucorrhœa, the source of innumerable diseases in women, and one which is too often overlooked or neglected.

The articles on Spinal Meningitis and Myelitis by Dr. Bennett, though brief, are judiciously written; and valuable, as forming some addition to the small number of treatises on these subjects which are accessible to the mere English reader. The subject of Hydrorachis succeeds, and is well handled by Dr. Bennett. A notice of Spinal Apoplexy, by the same writer, contains what little there is to say upon the subject.

The article on Chorea, by Dr. Theophilus Thompson, is an excellent one. The ordinary nosography of chorea as it now presents itself is followed by a short account of the singular modifications of it which have been observed at different times from the first outbreak of the dancing mania. These are divided by the writer into three classes: 1. Those consisting chiefly in energetic and often measured actions of the muscles under the influence of a morbidly excited will, as the dances of St. John and St. Vitus; tarantism; and the leaping ague of Scotland. 2. Those in which the movements are systematic but involuntary, as malleation;* rotation; and propulsion, forwards or backwards. 3. The convulsive disorder which has occasionally prevailed as an epidemic in times of religious fanaticism, and which is usually accompanied with irregular exacerbations and remissions.

Our author observes that the phenomena of the last class have been well described by Dr. Robertson, as they occurred among a sect of en-

* Striking of the knees with the hands as with a hammer.

thusiasts in Tennessee and Kentucky in the year 1800, (Inaug. Essay on Chorea St. Viti, 1805.) He mentions a similar affection as occurring at Cambuslang in the year 1742. Here the movements which were at first voluntary became spasmodic, the muscles of the neck and upper extremities were convulsed, and the sufferers were thrown down and agitated with motions like those of a fish on land. (p. 208.) The occasion of these performances was no other than the famous "Cambuslang awakening," the impression of which was long felt among the peasantry of the south of Scotland, and which is even now frequently talked of.

Some extraordinary developments of blended chorea and mania have at different times resulted from the supposed machinations of infernal spirits or their human agents. The curious reader may find some cases of this kind ludicrously exaggerated by superstitious credulity in Cotton Mather's Ecclesiastical History of New England.*

Dr. Thompson's remarks on the morbid anatomy and immediate seat of chorea are, we think, extremely good, and we regret that our space will not allow us to insert them. The general indications in the treatment of chorea are stated to be: 1, To ascertain whether there be any congestion or irritation of the cerebro-spinal axis, and if there be, to relieve it by moderate local depletion; 2, To act freely on the bowels by suitable purgatives; and 3, To administer remedies calculated to invigorate the frame, diminish nervous susceptibility, and increase the energy of the digestive function. (p. 213.) The particular means of fulfilling these indications are explained with judgment and precision.

The succeeding article on Hysteria is by the same writer. His description of the irregular forms of the disease is scarcely so full as we could have wished, and we think he might with advantage have dilated on some points of diagnosis. We may here, in passing, notice one circumstance not usually mentioned by writers, but which it is important to attend to in practice, namely, that hysterical pain in the abdomen and head is sometimes accompanied with an excited pulse and a hot skin. In one hysterical patient we have witnessed extreme tenderness of the abdomen, with a quick and full pulse, a hot skin, and frequent vomiting. In the same patient hysteria sometimes mimicked phrenitis, yet in both instances the physiognomy and manner of hysteria were so strongly marked as to leave no doubt of the real nature of the case on the mind of an attentive observer. This lady, however, when from home, more than once fell into the hands of practitioners who, misled by the symptoms, bled her, with great aggravation of the evils. Dr. Thompson is, we believe, quite correct in affirming that the cerebral excitement of hysteria is often mistaken for phrenitis, to the great prejudice of the patient. Acceleration of the pulse accompanying hysterical attacks arises, we apprehend, from an irritable state of the heart, and may be distinguished from febrile or inflammatory excitement by its not being uniform, but varying with slight changes in the state of the patient's mind, and other circumstances which would not affect the pulse of fever or inflammation.

* Magnalia Christi Americana; or the Ecclesiastical History of New England, &c.—London. Fol. 1702. Bk. vi. Ch. 7. Four children in particular were "arrested by a very stupendous *Witchcraft*."

There is perhaps no malady in the diagnosis of which we may derive more important aid from physiognomy than hysteria. Medical physiognomy is indeed in many diseases a source of diagnosis which seldom fails the practitioner who is intimately versed in it; and we believe that much of that exquisite tact in the discrimination of disease which distinguishes some practitioners, and which others can never attain, depends on the vivid perceptions of an eye and ear habitually familiar with the lineaments, the tones, and the gestures of disease.

On the whole, this article is a very able one. The directions for the treatment of hysteria are both copious and judicious, and the concluding paragraph on female education contains much truth happily expressed.

In the articles Tetanus and Hydrophobia, Dr. Bennett has given a good exposition of what is known concerning these diseases. We may remark, however, that we are extremely sceptical as to the cases of recovery from hydrophobia referred to by the writer; and we feel ourselves compelled to state that the very first of the cases alleged, in page 260, to have been cured by bloodletting, was not an example of the disease in question, and was not even adduced as such by Dr. Innes, by whom it is recorded. It is entitled, "An inflammation of the stomach, with hydrophobia and other uncommon symptoms." The disease is not referred to the bite of any animal, and the hydrophobia is only mentioned as a symptom. (Edin. Med. Essays and Obs., vol. i. p. 227; Ed. 1752.)

Dr. Theophilus Thompson has done great justice to the subject of Neuralgia, and that of Paralysis is ably treated by Dr. Bennett. We have nothing particular to say on any of these heads, nor on that of Barbiers, a short notice of which is given by Dr. Bennett.

The volume closes with three articles on Inflammation of the Eye, on Amaurosis, and on Otitis: the two former by Dr. Taylor, the last by Dr. Bennett. These we omit to notice because we have recently treated so largely on this class of diseases.

We trust we shall not be accused of a carping disposition if, before concluding, we point out some literary faults which detract from the general merit of this volume, and for which we hold the editor more responsible than the authors of the articles. "A *mutual* relation to *each other*," (p. 1,) and "*post-mortem* appearances found *after death*," are grievous tautologies. "The practitioner should not be *unwarily scared*," (p. 15), is a queer expression; and "suddenly-or-otherwise-injudiciously-checked morbid discharges" is considerably more in the genius of the German than of the English language. Many examples of equally bad writing might be adduced, and the defects of the English, we are sorry to say, are by no means compensated by the excellence of the Greek. At p. 231, the tetanic distortion of the body to one side is designated as "*pleurosthotonos*, from πλευροσθεν sideways." There is no such word as πλευροσθεν, and *pleurosthotonos*, though commonly enough used, is a barbarism; it should be *pleurothotonos*, from πλευροθεν, sideways. At p. 281, χωφος should be κωφος. In the same page, *ageustia* is derived "from α priv. and γευστος taste;" but γευστος is an adjective, and signifies capable of being tasted, i. e. having a flavour. But we hate the snarling part of our duty, and will therefore note no more minor faults. The volume taken altogether is highly creditable to those engaged in its production; many parts of it are written with great ability, and we have no hesitation in heartily recommending it as a valuable contribution to practical medicine.

Vol. III. In this volume are described diseases of the respiratory organs and passages, and of the heart.

The subjects of Bronchial and Pulmonary Diseases are treated of with considerable fulness and with the acknowledged precision of that physician, by Dr. C. B. Williams. However, as the different articles are scarcely more than reprints of a series of lectures already placed before the public in the Medical Gazette, it is unnecessary to undertake any very close examination of them on the present occasion.

Another instance of defective nomenclature strikes us in turning over these essays: Why does Dr. Williams speak of tracheitis *or* croup, when he himself labours to prove, what no one who has seen the disease will question, that this is not tracheitis in the ordinary acceptation of such term? We might add, in *any* acceptation; for, admitting that foundation exists for the notion advocated by Mr. Ryland and himself, that the cellular membrane as well as the mucous is engaged in the inflammatory action, there is besides this a nervous or spasmodic element in the disease, which separates it effectually from the whole class of maladies distinguished by the suffix *itis.* Now the importance of the condition of the laryngeal muscles is, practically speaking, extreme; for this is, in rare instances, the efficient cause of death. A case observed by Lobstein demonstrates this: the patient indubitably had true croup, for he had expectorated pseudo-membrane, yet, as dissection disclosed, not the least trace of lesion of any kind existed in the air-passages at the time of death.

Dr. Williams includes among the characters of an asthenic form of the disease, *extension* of the albuminous exudation to the throat and fauces. He neglects to notice the very important opinion, originally stated by Bretonneau, and subsequently espoused by numerous observers of eminence, that croup is frequently *preceded* by pseudo-membranous inflammation of the pharynx: M. Guersent estimates at nineteen out of twenty the number of cases of croup in the infant originating in this manner. We are perfectly aware that this statement will be received by many practitioners with stubborn incredulity; but of persons thus meeting the alleged fact, it may not be improper to enquire, in how many cases they are prepared to prove they have examined the fauces with the necessary care at the outset of the complaint.

The experience of Mr. Porter, M. Hervez de Chegoin, and others shows that the morbid exudation may *commence* in the bronchi, and thence spread upwards. Dr. Williams might also have supplied his readers with more accurate information respecting the frequency with which the plastic formation arises secondarily or otherwise in those tubes, than the vague intimation that it occurs "in some instances." M. Guersent publishes the following analysis of 150 cases by MM. Houssenot and Bretonneau:

| | |
|---|---|
| False membrane not extending below the bronchi . . . . | 78 |
| ,, ,, seated in the bronchi . . . . . . | 42 |
| ,, ,, in larynx or trachea, the state of the bronchi not being mentioned . . . . . . . | 30 |
| | 150 |

The history of croup in the adult surely merited separate consideration, though Dr. Williams has not acted as if he thought so.

"It is not necessay," says Dr. Williams, "to discuss the question of the propriety of resorting to tracheotomy in croup, as it has been decisively negatived by Dr. Cheyne, Mr. Porter, and others of the *best authorities.*" These eternal authorities! How shall truth ever force her way, while the dicta of these lawgivers suffice to suppress even discussion? But even assuming the question to be one of authority; who shall compare the respect due, on the one hand, to the assertion of MM. Bretonneau and Trousseau, who have performed the operation ninety-eight times, and on the other to that of Dr. Cheyne, who probably never cut into the trachea in his life? Now Bretonneau and Trousseau tell us to operate, and affirm that doing so affords in many cases the only hope of rescuing the patient from imminent death. "Authority" is therefore, we suspect, rather against Dr. Williams. But let us consider the question as one of facts and not of names. The facts, then, are that of 140* cases of croup in which the operation has of late years been performed by different French surgeons, 28 [20 per cent.] have terminated by recovery. Is this a small share of recoveries, when, as Dr. Williams reports from Double, the mortality in the present day† is nearly one half [50 per cent.] of the whole numbers attacked? formerly, when the treatment of the disease was less understood, it amounted to nearly four fifths." [80 per cent.] But further, let it be remembered that the vast majority of these cases were in an utterly hopeless state when the knife was used. What proportion of cases, we would ask, that have fallen into the utterly hopeless category, would recover independently of tracheotomy? Numerical precision cannot in the present state of our knowledge be given to any inference on this point; but we fear did this exist it would prove the deaths to be something very like 10 per cent. of the cases. It is probable that the main cause of failure in unfortunate cases is that the operation is habitually delayed too long; this is rendered indeed almost certain by the fact that while Bretonneau and Trousseau, who performed it at an earlier period than others, can boast of 25·4 per cent. of recoveries, their brethren can exhibit no more favorable result than 9·5 per cent. Another cause of failure is to be found in the existence of pneumonia, marked and extensive, at the period the section of the trachea was made.

Is the result of the operation for hernia performed *in extremis* vastly more favorable than from existing facts the operation for croup is proved to be?

* These numbers are taken from a table given in the article Bronchotomy, by Mr. Wells, in the Cyclopædia of Practical Surgery, vol. i., p. 513.

† Double's book was written before Bretonneau's performances had drawn the attention of observers to opening the trachea.

Influenza. This is written by Dr. Theophilus Thompson. Under the head of "nature of the disease," the author having observed upon the rarity with which uncomplicated influenza destroys life, states that in the rare instances when such event has occurred, the mucous membrane of the larynx and bronchi has been found of a deep red colour, flakes of lymph sometimes observed on the chordæ vocales and in the ventricles, the trachea found injected and lined with glassy-looking mucus, the lungs overcharged with sero-mucous fluid, engorged and even consolidated in a limited extent of their substance. His views of the essence of the malady appear in the following paragraphs: "The danger in extreme cases of influenza appears to arise from an excess of mucus preventing the due arterialization of the blood. The difficulty and rapidity of the respiration are, however, out of all proportion to the quantity of secretion, or even to the amount of inflammation, and the dyspnœa is sometimes intermittent; and this circumstance cannot easily be explained except by supposing that the cause of the disease must operate, by producing an impression on the vital energy of the lungs, analogous to that occasioned by cutting the nervus vagus; and we may reasonably conjecture that influenza depends on an influence exerted on the nervous system, especially on that part of it having most relation to the bronchial mucous membrane, tending to elicit any latent predisposition to disease, and modified in its character by varieties of constitution as well as by peculiarities of climate and other external conditions. . . . . . . This opinion derives support from the liability, so frequent in this complaint, to derangement in a great variety of organs, as well as from the occasional occurrence of inflammation of the spinal cord, and of inflammation or other affections of the brain. The effects of such a shock thus communicated to the nervous system may be expected to develope themselves in the weakest organ and to vary according to collateral circumstances. Thus, in the same epidemic, one patient will suffer from meningitis, another from enteritis, a third from rheumatic affections. If a sudden increase of temperature succeed to frost or snow, pneumonia will frequently be found associated with the complaint, whilst exposure to fatigue and mental anxiety will increase the liability to erysipelatous complications." (p. 207.)

Respecting the degree of severity of the English epidemic of 1836-7, Dr. Thompson states that "the deaths, as far as could be ascertained, were about two per cent. of the number attacked, a proportion corresponding with that deduced by Ozanam from a calculation of the mortality of all the recorded instances of epidemic catarrh." And he deduces from his own historical sketch that, "since medical records have become available, influenza has prevailed on an average once in ten years, and has proved the most destructive of epidemics." We presume that Dr. Thompson means that the influenza has, in the gross, destroyed most lives; as it is impossible he could have intended to term an epidemic cutting off two per cent. of those seized the "most destructive" of that class of maladies.

The point mooted in the following sentences is not unworthy of consideration:

"There is also reason to believe that a modified condition of the atmosphere may remain for years after the prevalence of the disease, and occasion a liability to affections of a similar character, to which the term *influenzoid* might be applied. For eight years after the prevalence of influenza at Lyons, this was found to be the case: 1300 deaths out of 10,096 occuring during that period being attributed to catarrhal or mucous fevers. We are inclined to believe that a similar condition has existed in this country since 1833, affections of the bronchi and fauces having been unusually prevalent, associated with severe muscular pains and unusual depression of strength." (p. 207.)

Dr. Thompson seems much too regardless of the importance of accurate bibliographical references; what is the value of an intimation that certain cases are related in "Rust's Mag. and Lond. Med. Gaz.," without even their author's name being mentioned? Does Dr. Thompson mean to "laugh at the beards" of those who might feel inclined to enquire into his accuracy as a reporter of the opinions of others?

The essay on Asphyxia, by Dr. Carpenter, contains a very complete account of all that is actually, or is believed to be, established of the practice respecting that condition. The article is written with great clearness and precision, and the subject generally treated with much comprehensiveness; but as it professes to contain nothing particularly novel, we shall content ourselves with recommending its perusal to our readers.

Dr. Joy has contributed a very excellent essay on Diseases of the Heart, exhibiting correct acquaintance with recent additions to our knowledge of this class of maladies. We have rarely met with a monograph having stronger claims to attentive perusal. Nevertheless, we cannot agree with all Dr. Joy's opinions. Thus, he quotes with apparent commendation the estimate of Bouillaud, that "at least one half of all the cases of acute rheumatism occurring in practice, or what is ordinarily called rheumatic fever, are accompanied in some part of their course by an inflammation either of the internal or external lining of the heart, or both;" whereas we are persuaded, and we know that many close observers unite in this persuasion, the assumed proportion is very materially too high. Further, M. Bouillaud maintains, as our readers are aware, that the inflammation of the membranes of the heart commonly proceeds *pari passu* with the articular affection, instead of, as originally stated by English writers, arising in consequence of metastasis from the joints to the heart. Dr. Joy thinks M. Bouillaud underrates the frequency of the latter mode of development; a correction in the justness of which we are the less disposed to coincide, because we find Dr. Joy seeking an *à priori* argument in its favour in the similarity of the tissues engaged in the inflammatory process in both situations. Why should not this similitude be as fair a reason for their suffering *simultaneously?* In fact, we long since heard M. Bouillaud assert that it was such, and affirm that the identity of structure would suffice to show that both orders of tissue *must* frequently be affected together;* a mode of prejudging the question which first led us to conceive a doubt, justified and confirmed by subsequent experience, of M. Bouillaud's perfect accuracy in relation to the point already referred to. The truth is that identity or non-identity of

* So are the arachnoid and tunica vaginalis both composed of the same species of tissue; yet M. Bouillaud will hardly affirm that individuals with meningitis are especially liable to have inflammatory hydrocele.

tissue has nothing to do with the matter; the question is one to be decided by direct observation alone.

We are pleased to observe that Dr. Joy has given M. Louis his due position among the successful investigators of pericarditis. He it was that first placed its diagnosis within the reach of the ordinarily attentive observer by his just discrimination of the three most important physical signs of pericarditic effusion; unnatural dulness under percussion of the præcordial region, vaulted form of that region, and distance and feebleness of the cardiac sounds. But it has been the habit to forget M. Louis' modest memoir in admiration of the *tomes à prétention* of others.

Here is a nice point in diagnosis which we can readily conceive might become of importance at the bedside:

"An enlarged and feebly-acting heart may be distinguished from the case of profuse pericardial effusion with weak impulse and distant and indistinct sounds, by applying the stethoscope to the supra-clavicular region, where in the latter case, and in it only, the cardiac sounds will be heard with considerable clearness in the course of the carotid and subclavian arteries, indicating that their feebleness in the præcordial region is the result of an obscured, rather than of an actually deficient action." (p. 318.)

We extract a portion of the account of the *bruit de frottement* of pericarditis, as affording a useful practical lesson and a good specimen of Dr. Joy's manner:

"Very early in the course of the disease, as on the second or third day for instance, a faint rubbing or rustling sound (bruit de frottement, or to-and-fro-sound, murmur of ascent and descent, &c.) such as that produced by the friction of silk, paper, or parchment, is frequently audible, accompanying both sounds of the heart. It gradually assumes a louder rougher character, and generally extends eventually over the whole region of the heart, and materially obscures the natural sounds of the organ, though they may still be recognized by applying the stethoscope near the top of the sternum. It has its source in the friction of the opposed surfaces of effused lymph, which, even whilst still very thinly spread and soft, is quite sufficient for its production, as has been fully ascertained by the experiments of Drs. Williams, Clendinning, and Todd. Whether the first stage or that of simple congestion and dryness of the membrane be capable of giving rise to it in a minor degree is still doubtful; the experiments of the gentlemen just named render it, indeed, very improbable that it is ever heard except in those cases where ecchymosis under the pericardium, or some slight traces of coagulable lymph on its polished surface, already exist.

"The rubbing sound occasionally somewhat changes its character, and becomes perfectly similar to the creaking of leather in the sole of a new shoe or saddle (cri de cuir, leather-creak). This was first observed by M. Collin; and though Laennec was latterly sceptical as to its import or reality, it has been since fully confirmed as a valuable sign of pericarditis by Stokes, Reynaud, Watson, Mayne, Bouillaud, Williams, and others. . . . . . The peculiar sound in question is commonly distinguishable from that connected with valvular disease, as Dr. Stokes many years ago pointed out, by the suddenness of its occurrence, and by the short distance from the cardiac region within which it is audible, as well as by the greater influence of treatment over it. But still there may be considerable difficulty of diagnosis where disease of the valves has preexisted, or where endocarditis springs up simultaneously. The rubbing sound is, however, of a decidedly more superficial and equally diffused character than the bellows-murmur, indicative of disease of the valves and orifices; and is more constantly double, or an accompaniment of both motions of the heart." (pp. 312-3.)

Vol. IV. The fourth volume commences with the diseases of arteries and veins. These are illustrated by Dr. Joy under the heads of functional diseases of the arteries; arteritis; aneurism of the aorta; phlebitis; varicose veins; miscellaneous affections of the veins, &c.

In allusion to *neuralgia* of the arteries, the author expresses himself, we think, in too general terms concerning the distribution of the sympathetic nerve on the coats of these vessels. He says that the seat of the pain is most probably in the minute ramifications of the ganglionic nerves which form a close network around the arterial trunks, and penetrate into the substance of their walls. (p. 1.) Now, the anatomical statement here made is true with respect to the arteries of the head, neck, thorax, abdomen, and organs of generation; but its truth is very doubtful with respect to those of the extremities. It is singular, indeed, that so important a point in physiological anatomy should have been so little the subject of practical enquiry; for although it has been believed by many, and stated as a direct result of observation by some, that the ramifications of the sympathetic accompany the subdivisions of the arteries throughout the body, till they elude the senses by their tenuity, we are not aware that a single anatomist has laid before the public any details of the dissections by which he has been led to such a conclusion.

The subjects of abdominal pulsation, aneurism of the aorta, phlebitis, and other affections of the veins are judiciously handled; the author, however, appears to have derived his materials rather from reading than from personal observation, and consequently these articles afford little scope for criticism. We believe him to be right in withholding his assent from the doctrine now so prevalent that *phlegmasia dolens* is exclusively dependent on phlebitis.

"Many pathologists, however, are still averse, and we think with reason, to ascribing such an extensive influence to the veins in the production of the swelled leg of lying-in women, believing that they are only implicated in common with or even subsequent to several other tissues, more especially the subcutaneous cellular membrane, the inferior surface of the cutis vera, and the superficial nerves; for the swelling here, unlike that induced by the ligature of a vein, most frequently begins in the groin, labium, and thigh, and afterwards spreads downwards, in place of always manifesting itself first in the distal extremity of the limb; and it is accompanied, moreover, by an acute neuralgic tenderness diffused over the whole surface, and not met with in the same degree in cases of pure phlebitis, in which the pain and sensibility to pressure are more localized in the course of the vessels. Again, in phlegmasia dolens, the swelling is not œdematous, but from the quantity of coagulable lymph poured out it is tense and elastic, and when the disease is fully formed, rises immediately after pressure, and cannot be evacuated in almost any degree by puncture or incision; and finally the type of the accompanying fever is very dissimilar to that in indisputable phlebitis, and the rate of mortality is incomparably lower." (p. 29.)

The diseases of the organs of digestion succeed, and, taken a sa whole, are extremely well treated. The following articles are all by Dr. Symonds: stomatitis; gangræna oris; diseases of dentition; glossitis; parotitis; angina; hypertrophy of the tonsils; diseases of the œsophagus; gastritis; organic diseases of the stomach; dyspepsia; gastrorrhœa; inflammation of the duodenum; enteritis; inflammation of the cæcum; diarrhœa; cholera; alvine concretions; hemorrhois; spasmodic stricture of the rectum; colic; lead colic; torpor of the colon; tympanitis; peritonitis; enteralgia. The diseases of the mesenteric glands, and of the assistant chylopoietic viscera, are commented on by Dr. W. Thomson.

In treating of Stomatitis, Dr. Symonds has done well to notice, after M. Guersent, that an epidemic aphthous fever sometimes prevails in Holland, the most frequent subjects of which are adults, and especially puerperal women. (p. 36.) This disease, though curious, has escaped the attention of the greater part of medical writers, which perhaps has arisen from the small number of medical works published of late years by the practitioners of Holland and Flanders, throughout which countries the malady in question appears to have prevailed time immemorial. It is generally attributed by the authors who have described it to the cold and moist atmosphere of those regions.* We notice the subject because we conceive the study of all epidemic and endemic diseases to be of the highest importance to the etiology of disease in general.

When about to speak of the diseases incident to the process of *dentition* (p. 40), Dr. Symonds very properly premises that this process is one of *development*; a truth too little familiar to pathologists, but which has been placed in a prominent point of view by M. T. G. St. Hilaire, in his excellent work on the Anomalies of Organization. Impressed with the correctness of his notions, we feel convinced that those who refer the various diseases incident to dentition merely to the local irritation in the gums, and the sympathetic disorder of the intestinal or other surfaces, take too limited a view of the subject. Fully admitting the influence of these local causes, we conceive that dentition will also vary in its phenomena, and be more or less favorable in its progress, in proportion as the powers of the constitution are more or less equal to the development of a new set of organs.

In this respect it bears an analogy to puberty. It is true that the relations of general physiology to the theory of medicine are extremely vague in the present state of our knowledge; but they should not on that account be left out of consideration; on the contrary, it should be our ceaseless endeavour to render them more distinct, for herein lies the hope of approximating medicine to the exact sciences.

The subject of Gastritis is introduced by some very good general remarks on the anatomical characters of congestion and inflammation of the gastro-enteric membrane, (pp. 54-7,) and the observations on the physiological relations of the stomach which precede the consideration of *dyspepsia*, (pp. 71-3,) are also well worthy of attention. We quite agree with Dr. Symonds as to the propriety of considering dyspepsia apart from gastritis, admitting at the same time that chronic gastritis constitutes one of the forms of what is called dyspepsia. The following remarks appear to us to be highly judicious:

* The earliest account of it with which we are acquainted is that by Vincent Ketelær, entitled "Commentarius medicus de Aphthis nostratibus, seu Belgarum Sprouw. Auctore Vincentio Ketelær, Med. Doct." 12mo. Ludg. Batav. 1672. Dr. Craigie, in his Practice of Physic, places this work at the head of the bibliographical list relating to *thrush;* but he probably had not seen the work itself, as the exact title is not given, and his own remarks on epidemic thrush refer to it solely as a disease of infancy; whereas Ketelær describes this disease as common to all ages, and both sexes.

"Some authors consider dyspepsia as alway dependent upon an inflammatory or congested state of the mucous membrane of the stomach. This, however, we consider to be a narrow view of the subject, and long observation has led us to believe in the existence of a purely functional disorder of the stomach, that is uncomplicated with any structural alteration or with appreciable permanent disease of the capillary circulation. Of almost every other organ the same remark obtains, certainly of the brain, the lungs, the liver, and the kidneys. But while we maintain that the collective symptoms resulting from chronic gastritis constitute only one form of dyspepsia, we concede the difficulty, in a great many instances, of pronouncing a similar set of symptoms to be independent on such a state of the mucous membrane, and also that great errors in practice are committed every day by overlooking this frequent cause of a disorder, which by many is treated as if it were always functional. Let us endeavour to point out one or two features, more especially characteristic of the cases in which an inflammatory condition prevails. Pain spontaneous, or occurring after food, may depend on mere increase of sensibility. It is often concluded that the pain is not inflammatory if relieved by stimulants and carminatives; but this is not decisive for reasons which will appear when we discuss the treatment, though in the majority of cases of gastritis, the pain would be aggravated by such means. We have found a better test in the effect produced by hot liquids, such as tea or plain water, which seldom fail to aggravate or induce pain in these cases. The existence of tenderness at the epigastrium will confirm this evidence, but cannot be alone relied upon. The state of the tongue used to be thought one of the strongest diagnostic signs, but is liable to great fallacies. Andral, (Clin. Med., t. iv.) and Louis (Gastro-ent. t. ii. p. 64,) have proved that gastritis may coexist with a moist clean tongue of a natural colour; and on the other hand that this organ may be red, papillated, or even aphthous, with a healthy state of the stomach. Still in a large proportion of cases, such alterations of the natural appearance of the tongue as we have enumerated among the symptoms are observable, and should at all events lead us to suspect the disease. The state of the skin is an important help to us; thus squamous and papular disease, coexisting with stomach disorder, intimates very strongly that the mucous membrane is inflamed. The relief afforded by antiphlogistic means affords some useful hints; but it must be valued only in connexion with other signs. We may remark, however, that relief ensuing upon iced drinks is more decisive than when produced by local depletion, for the latter will sometimes mitigate a purely nervous gastralgia. The nature of the matters vomited is a valuable indication. For example, it is improbable that a large quantity of mucus should be secreted, unless the membrane had been previously in a state of plethora." (pp. 59-60.)

The subjects of ENTERITIS and CHOLERA are well handled; but as we have nothing particular to say relating to them we pass them by.

Dr. Symonds mentions two cases of NON-MALIGNANT STRICTURE OF THE COLON presenting varieties not hitherto described by authors. In one of them the stricture was situated in the arch of the colon, and appeared to have been caused by a folding inwards of nearly half an inch of the intestines, with hypertrophy of the mucous and sub-mucous tissue of the part, originally caused, as the author thinks probable, by a small invagination which became permanent by adhesive inflammation. In the other case the stricture, which barely admitted the point of the little finger, was seated just above the termination of the colon in the rectum, and was caused by hypertrophy of the adipose tissue under the serous coat. (p. 123.) We have not space to give the descriptions in full.

The article on Hemorrhois is a good and practical one. With respect to simple hemorrhage from the rectum the writer remarks:

"A frequent cause, though often overlooked, is a small vascular point on the surface of the mucous membrane, from which a minute artery throws a jet of blood every time the bowel is evacuated. This may be unfelt, and the hemorrhage may occur daily, unknown to the patient or his medical adviser, till general anæmia awakens a suspicion of the true nature of the disease." (p. 126.)

We are surprised that Dr. Symonds having made this remark should not have noticed that the hemorrhage which accompanies internal piles is usually not venous, as might be expected, but arterial. This fact has been stated by Sir B. Brodie, and we have had frequent occasion to verify it. We agree with Dr. Symonds that hemorrhage from rupture of the vein, forming a hemorrhoidal tumour, is of unfrequent occurrence. Indeed, according to our observation, the hemorrhage, when venous, is for the most part trifling, and consists in a mere exudation; when it is at all profuse it is arterial, and proceeds from within the rectum.

We are not aware of any point which calls for particular notice in the other essays by Dr. Symonds, of which we have enumerated the titles, unless, indeed, it be one relative to the treatment of peritonitis. The writer states that he has tried the experiment of placing the patient in a warm bath sufficiently long and shallow for him to lie extended with the tumid abdomen rising above the level of the water, and pouring cold water upon the abdomen. He tells us that the relief of pain has been striking even where the disease was too far advanced to admit of cure. (p. 145.) We know not what benefit may be derived from the application of cold to the abdomen while the blood is drawn to the surface of the rest of the body by the action of heat and moisture; but under ordinary circumstances we are no advocates for the external application of cold in abdominal inflammation, the few trials we have made of it having been so discouraging as to prevent their repetition.

The article on Diseases of the Mesenteric Glands is admirable. Dr. William Thomson has here borrowed largely (with due acknowledgment) from the valuable article *Carreau*, by M. Guersent, in the Dictionnaire de Médecine, and has added what was profitable from the writings of British pathologists. He discriminates properly between simple inflammatory enlargement and tubercular disease of the mesenteric glands; very distinct cases which some eminent practitioners have lately confounded in a manner to us unaccountable.

The same author treats of the Diseases of the Liver and Biliary Organs; and, as the article has been republished in an enlarged form, we have noticed it elsewhere.

The Diseases of the Pancreas and Spleen succeed, and are descanted on by the same writer as lucidly as the unfrequency of disease in the former organ and our ignorance of the functions of the latter would admit.

The Diseases of the Urinary Organs are illustrated by Dr. Christison, and his name affords sufficient assurance that the articles relating to them must be written with ability. The opinions of the writer here expressed on that part of the subject which has lately occupied the most prominent place in the discussions of pathologists, granular disease of the kidney, are identical with those contained in his recent work, which we reviewed in a late number; and as the whole class of urinary diseases have frequently occupied our attention in this Journal, we will omit them altogether for the present.

The Diseases of the Uterine System are treated of under the heads of disordered menstruation; hysteralgia; leucorrhœa; inflammation of the uterus; polypus of the uterus; cauliflower excrescence; carcinoma; corroding ulcer of the uterus; other morbid degenerations of its structure; inflammation of the ovary; dropsy of the ovary; structural diseases of the ovary.

The first three of these are commented on by Dr. Ferguson, and the rest by Dr. Simpson. The papers of both these gentlemen are written with good sense and perspicuity, and in the off-hand manner of men who thoroughly understand their subject. With this general commendation we must pass them by, in order that we may take some notice of the next volume.

Vol. V. This volume contains dissertations on hemorrhage, with the allied subject of scurvy; on dropsy; scrofula; bronchocele; rheumatism; gout; and invermination: it concludes with a treatise on the art of prescribing, accompanied by a formulary. The subject of hemorrhage is introduced by some general doctrines, which merge, rather too abruptly perhaps, in the particular consideration of epistaxis; hemoptysis; hematemesis; hemorrhage from the intestines; hematuria; and hemorrhage from the uterus. These articles are by Dr. George Burrows, and are all good. We think, however, he has committed a mistake in that portion of the paper on uterine hemorrhage which related to the gravid state. "Uterine hemorrhage," he says, "may occur during pregnancy: this accident may happen in the early or in the more advanced stage of utero-gestation. When uterine hemorrhage occurs at the early period of pregnancy it is occasioned by the partial separation of the placenta from the uterus, and the probability of arresting the hemorrhage and preventing abortion will depend upon the extent to which the detachment of the placenta has proceeded." (p. 56.)

The assertion that hemorrhage occurring early in gestation is occasioned (exclusively we are led to infer) by partial separation of the placenta, seems to us not justified either by experience or authority. On the contrary, it is chiefly in the early stages that we meet with hemorrhage between the uterus and the epichorion; occasionally in the substance of the placenta; and, more rarely, between the membranes of the ovum.*

The article on Scurvy, by Dr. Budd, is ably written, and contains nearly all that is known on the subject.

* M. A. C. Baudelocque states that effusion of blood between the uterus and epichorion "is very common in the early periods of gestation, and that the greater part of abortions at these periods are attributable to no other cause." Traité des Hémorrhagies Internes de l'Uterus. (p. 35.) Ed. Bruxelles, 1832.

The succeeding articles, on Dropsy, are by Dr. Watson, and do credit to his judgment. That on the general doctrines of dropsy is particularly valuable, and the acute and febrile forms of the disorder are therein placed in an intimate, and we believe a true relation to disease of the kidneys. The observations of the writer on that variety which has been called acute anasarca, as well as those on dropsy following scarlet fever, are worthy of careful perusal, and we regret that our limits will not allow us to transcribe them. Dr. Watson's view of both cases is based on the supposition that the functions of the skin being primarily deranged, those of the kidney become so in consequence of the close relation which renders the latter complemental of the former; and that albuminous urine and the other conditions of renal dropsy are hence induced. (pp. 131-34.)

The subjects of Chronic Hydrocephalus, Thoracic Dropsy, and Abdominal Dropsy, are well and practically discussed. We are happy to find the writer boldly advocating paracentesis in those cases of chronic hydrocephalus to which it is applicable. This operation seems to us to have met with unmerited proscription from a number of writers. If we examine the cases on record in which it has been tried, we shall find that, in about one half, it is stated to have been successful. Now, making allowance for the fallacy of medical testimony in wonderful cases, which we are fully inclined to do, we will estimate the successful cases at one third, and ask if any other remedy in this disease has been equally fortunate? We might ask also if paracentesis has been as successful in dropsy of either of the other great cavities as in that of the head?

The chapter on Scrofula, by Dr. Shapter, is well written, and gives a copious and accurate view of the subject. It is chiefly a compilation, but this is not derogatory from its merits, since there have been no recent additions to our knowledge which could justify the introduction of new speculations.

On Bronchocele, all that is needful is said by Dr. Rowland. The point of chief interest with respect to this disease is its endemic cause. Dr. Rowland concludes, we believe justly, that it is yet to be discovered. To Mr. M'Clelland's view of the origin of bronchocele, so fully noticed in a former number of this Journal, (vol. VIII. p. 103,) in the use of water passing through limestone rocks, he objects: 1, That it is met with abundantly in districts where there is no limestone, as in the Vallais. 2, That it is absent in many places where limestone abounds. 3, That it prevails in districts where the waters are free from any calcareous impregnation. 4, That a strict adherence to the use of distilled water does not ward off the disease where it is endemic. He nevertheless admits that the use of calcareous waters strongly predisposes to the disease. He thinks also that other causes have the same tendency, though few in an equal degree; and that some influence, hitherto unknown, is necessary to the development of the disease.

The subjects of the remaining papers in this volume are not of a kind to be entered into profitably in a general notice like the present.

Rheumatism and Gout are well illustrated by Dr. W. Budd. With reference to the latter affection he is inclined to a humoral pathology, and congratulates himself in a note at the end of the article on the coincidence, in many points, of his own views with those propounded by Dr. Holland in his Medical Notes and Reflections, respecting the nature of gout as a disease of the blood, and the mode of operation of colchicum. These views, as we have elsewhere stated, we consider as worthy of particular attention, and as affording a very enticing subject of experimental enquiry to the iatrochemist.

The article on Worms, by Dr. Arthur Farre, is an exceedingly well-written and complete essay; we wish, however, that the author had not perpetrated, nor the editor admitted, such a word as verminology, (p. 257.) Why displace the ordinary term *helminthology* for so nasty-sounding a hybrid? The reader will not expect that we should here enter into the natural history of the entozoa, or the symptoms and treatment of the diseases occasioned by their presence.

The concluding essay, on the Art of Prescribing, by Dr. Joy, contains a good view of the operation of the different classes of medicines and of the rules to be observed in their administration: the formulæ also are well selected.

In taking leave of that portion of the "Library" which relates to the practice of physic, we have much pleasure in saying that, although we have found some fault, and might have found more if we had been ill-naturedly disposed, we regard this work as one of great value to the practitioner; and there is one respect in which it is especially so;—namely, in the full and precise directions which are given as to the manner of administering the various remedies recommended; a point on which the greater part of works on the practice of medicine are, in our opinion, extremely deficient. We must, at the same time, give it as our opinion, in justice to the authors, the editors, and the publishers of the Cyclopædia of Practical Medicine, that the present work is, in several respects, inferior to it, and certainly was not required by the profession so long as its predecessor could be procured in the shops; and we think that a more acceptable service would have been rendered to medical readers by the republication of the Cyclopædia, with such modifications and improvements as the lapse of time since its appearance and more experience on the part of its editors might suggest.

Our pained and wearied eyes at the conclusion of our arduous task of examining these volumes too well justify our former reprobation of their mechanical qualities. We repeat, that the long lines, the small type, and the paper neither smooth nor white, seem as if chosen on purpose to deter every reader, except the youthful student, from their perusal; and indicate at once a niggardly spirit and want of taste, which we shall leave to the editor and the publisher to share and appropriate as they best can.

